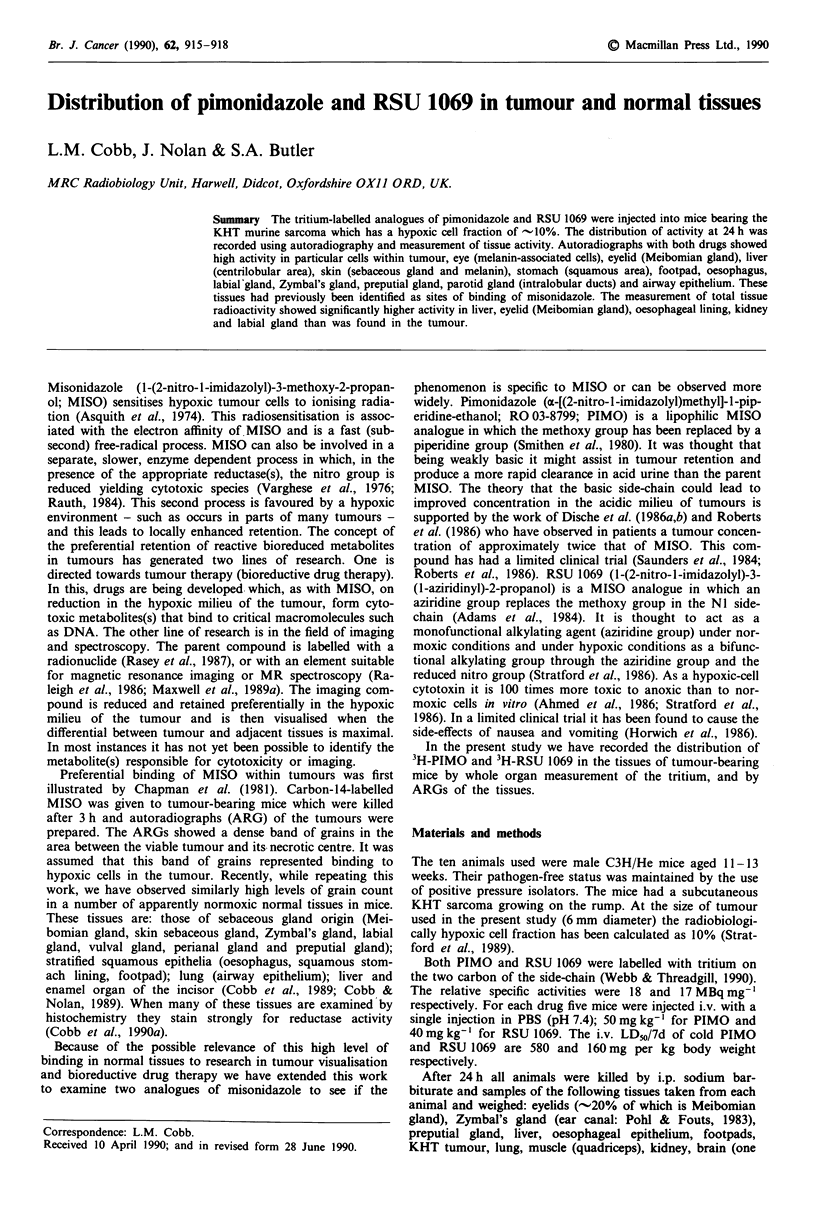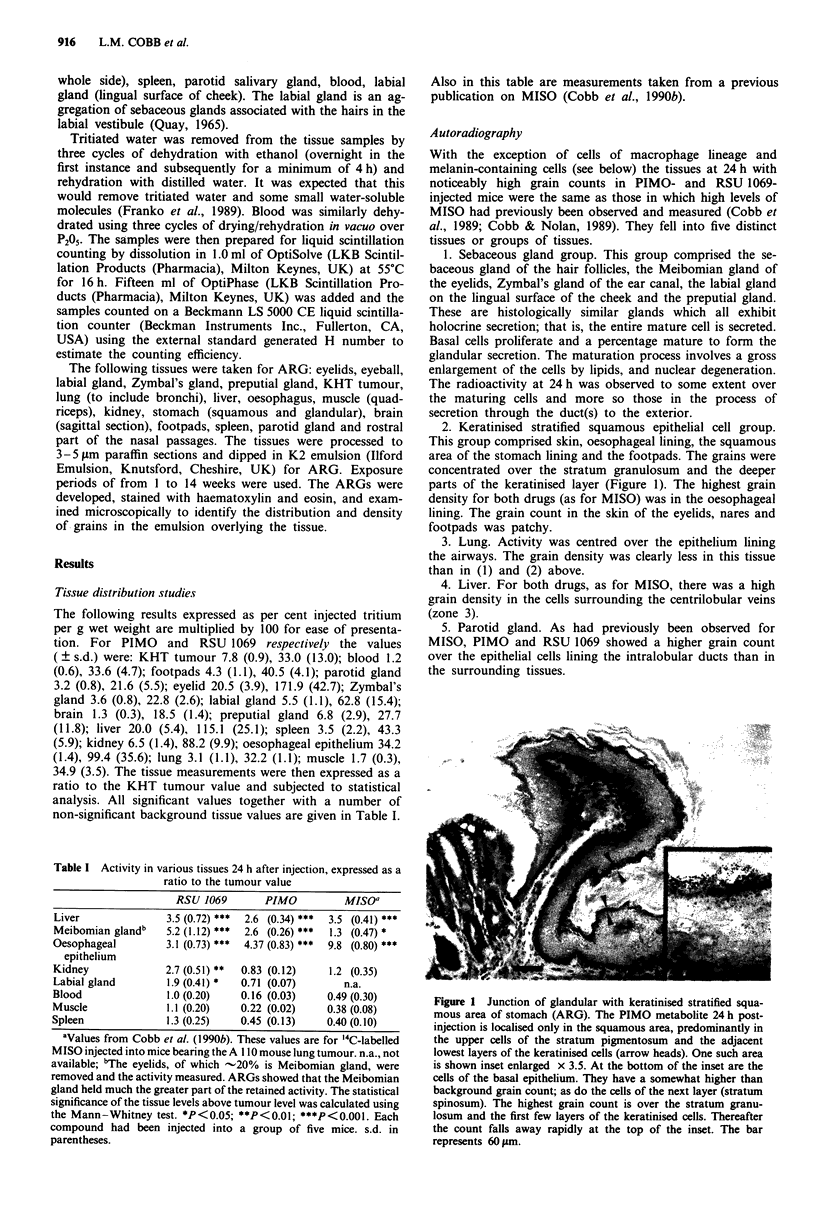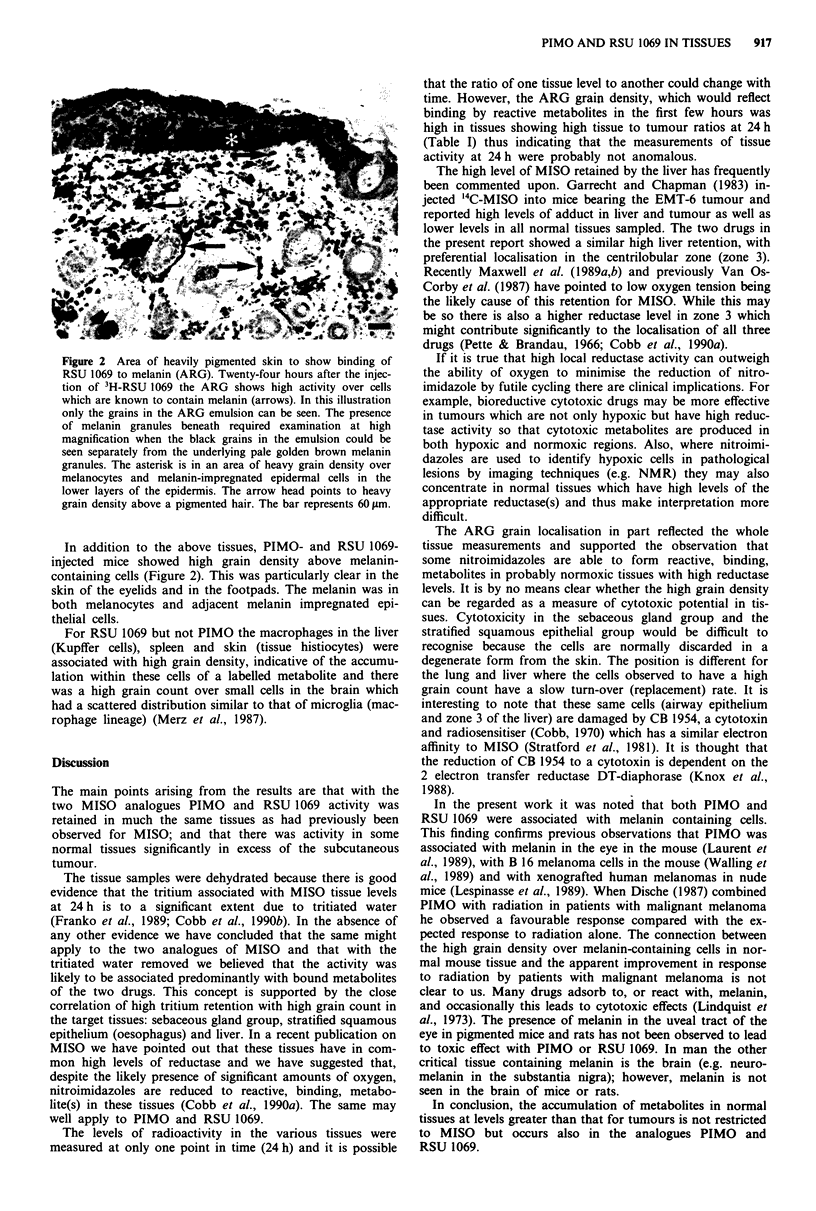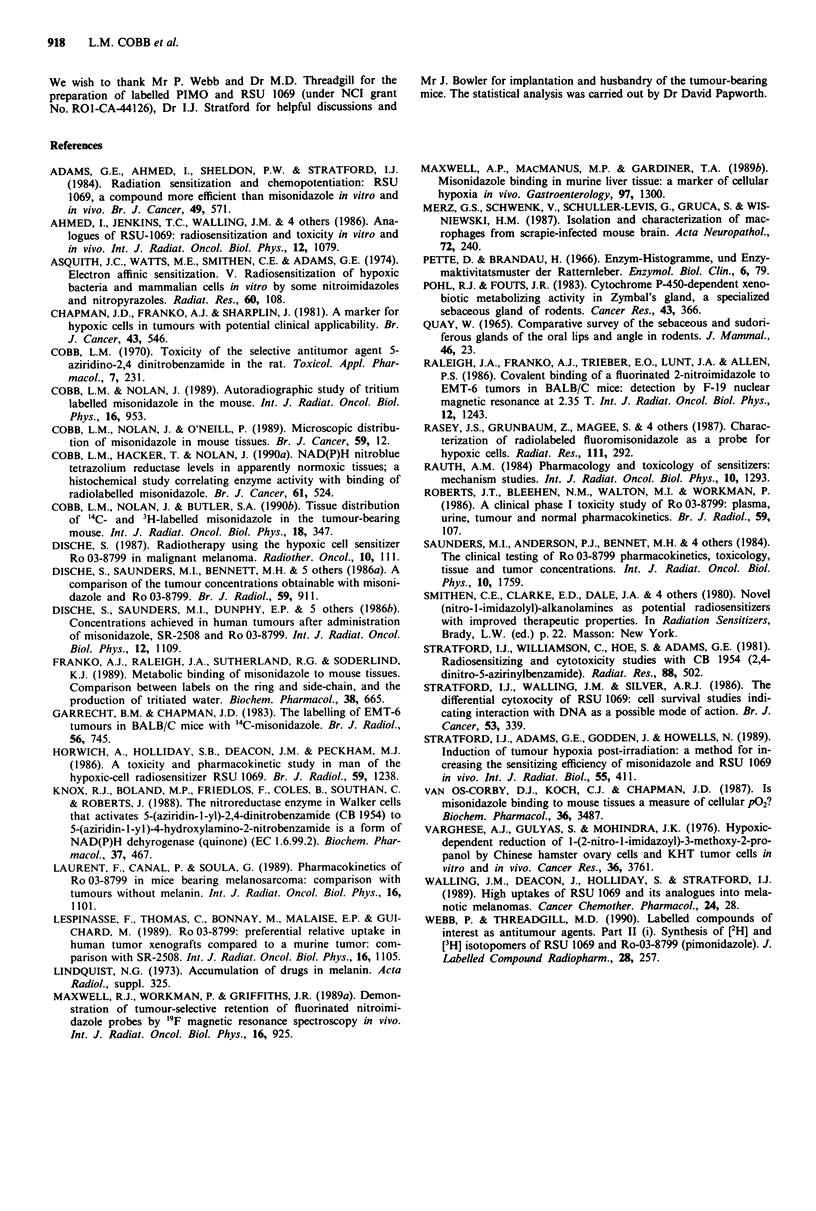# Distribution of pimonidazole and RSU 1069 in tumour and normal tissues.

**DOI:** 10.1038/bjc.1990.408

**Published:** 1990-12

**Authors:** L. M. Cobb, J. Nolan, S. A. Butler

**Affiliations:** MRC Radiobiology Unit, Didcot, Oxfordshire, UK.

## Abstract

**Images:**


*Br. J. Cancer* (1990), **62**, 915–918 © Macmillan Press Ltd., 1990

### Distribution of pimonidazole and RSU 1069 in tumour and normal tissues

L.M. Cobb, J. Nolan & S.A. Butler

*MRC Radiobiology Unit, Harwell, Didcot, Oxfordshire OX11 ORD, UK.*

**Summary** The tritium-labelled analogues of pimonidazole and RSU 1069 were injected into mice bearing the KHT murine sarcoma which has a hypoxic cell fraction of ~10%. The distribution of activity at 24 h was recorded using autoradiography and measurement of tissue activity. Autoradiographs with both drugs showed high activity in particular cells within tumour, eye (melanin-associated cells), eyelid (Meibomian gland), liver (centrilobular area), skin (sebaceous gland and melanin), stomach (squamous area), footpad, oesophagus, labial gland, Zymbal's gland, preputial gland, parotid gland (intralobular ducts) and airway epithelium. These tissues had previously been identified as sites of binding of misonidazole. The measurement of total tissue radioactivity showed significantly higher activity in liver, eyelid (Meibomian gland), oesophageal lining, kidney and labial gland than was found in the tumour.

Misonidazole (1-(2-nitro-1-imidazolyl)-3-methoxy-2-propanol; MISO) sensitises hypoxic tumour cells to ionising radiation (Asquith *et al.*, 1974). This radiosensitisation is associated with the electron affinity of MISO and is a fast (subsecond) free-radical process. MISO can also be involved in a separate, slower, enzyme dependent process in which, in the presence of the appropriate reductase(s), the nitro group is reduced yielding cytotoxic species (Varghese *et al.*, 1976; Rauth, 1984). This second process is favoured by a hypoxic environment – such as occurs in parts of many tumours – and this leads to locally enhanced retention. The concept of the preferential retention of reactive bioreduced metabolites in tumours has generated two lines of research. One is directed towards tumour therapy (bioreductive drug therapy). In this, drugs are being developed which, as with MISO, on reduction in the hypoxic milieu of the tumour, form cytotoxic metabolites(s) that bind to critical macromolecules such as DNA. The other line of research is in the field of imaging and spectroscopy. The parent compound is labelled with a radionuclide (Rasey *et al.*, 1987), or with an element suitable for magnetic resonance imaging or MR spectroscopy (Raleigh *et al.*, 1986; Maxwell *et al.*, 1989*a*). The imaging compound is reduced and retained preferentially in the hypoxic milieu of the tumour and is then visualised when the differential between tumour and adjacent tissues is maximal. In most instances it has not yet been possible to identify the metabolite(s) responsible for cytotoxicity or imaging.

Preferential binding of MISO within tumours was first illustrated by Chapman *et al.* (1981). Carbon-14-labelled MISO was given to tumour-bearing mice which were killed after 3 h and autoradiographs (ARG) of the tumours were prepared. The ARGs showed a dense band of grains in the area between the viable tumour and its necrotic centre. It was assumed that this band of grains represented binding to hypoxic cells in the tumour. Recently, while repeating this work, we have observed similarly high levels of grain count in a number of apparently normoxic normal tissues in mice. These tissues are: those of sebaceous gland origin (Meibomian gland, skin sebaceous gland, Zymbal's gland, labial gland, vulval gland, perianal gland and preputial gland); stratified squamous epithelia (oesophagus, squamous stomach lining, footpad); lung (airway epithelium); liver and enamel organ of the incisor (Cobb *et al.*, 1989; Cobb & Nolan, 1989). When many of these tissues are examined by histochemistry they stain strongly for reductase activity (Cobb *et al.*, 1990*a*).

Because of the possible relevance of this high level of binding in normal tissues to research in tumour visualisation and bioreductive drug therapy we have extended this work to examine two analogues of misonidazole to see if the phenomenon is specific to MISO or can be observed more widely. Pimonidazole (α-[(2-nitro-1-imidazolyl)methyl]-1-piperidine-ethanol; RO 03-8799; PIMO) is a lipophilic MISO analogue in which the methoxy group has been replaced by a piperidine group (Smithen *et al.*, 1980). It was thought that being weakly basic it might assist in tumour retention and produce a more rapid clearance in acid urine than the parent MISO. The theory that the basic side-chain could lead to improved concentration in the acidic milieu of tumours is supported by the work of Dische *et al.* (1986*a,b*) and Roberts *et al.* (1986) who have observed in patients a tumour concentration of approximately twice that of MISO. This compound has had a limited clinical trial (Saunders *et al.*, 1984; Roberts *et al.*, 1986). RSU 1069 (1-(2-nitro-1-imidazolyl)-3-(1-aziridinyl)-2-propanol) is a MISO analogue in which an aziridine group replaces the methoxy group in the N1 side-chain (Adams *et al.*, 1984). It is thought to act as a monofunctional alkylating agent (aziridine group) under normoxic conditions and under hypoxic conditions as a bifunctional alkylating group through the aziridine group and the reduced nitro group (Stratford *et al.*, 1986). As a hypoxic-cell cytotoxin it is 100 times more toxic to anoxic than to normoxic cells *in vitro* (Ahmed *et al.*, 1986; Stratford *et al.*, 1986). In a limited clinical trial it has been found to cause the side-effects of nausea and vomiting (Horwich *et al.*, 1986).

In the present study we have recorded the distribution of $^{3}$H-PIMO and $^{3}$H-RSU 1069 in the tissues of tumour-bearing mice by whole organ measurement of the tritium, and by ARGs of the tissues.

### Materials and methods

The ten animals used were male C3H/He mice aged 11–13 weeks. Their pathogen-free status was maintained by the use of positive pressure isolators. The mice had a subcutaneous KHT sarcoma growing on the rump. At the size of tumour used in the present study (6 mm diameter) the radiobiologically hypoxic cell fraction has been calculated as 10% (Stratford *et al.*, 1989).

Both PIMO and RSU 1069 were labelled with tritium on the two carbon of the side-chain (Webb & Threadgill, 1990). The relative specific activities were 18 and 17 MBq mg$^{-1}$ respectively. For each drug five mice were injected i.v. with a single injection in PBS (pH 7.4); 50 mg kg$^{-1}$ for PIMO and 40 mg kg$^{-1}$ for RSU 1069. The i.v. $LD_{50}$/7d of cold PIMO and RSU 1069 are 580 and 160 mg per kg body weight respectively.

After 24 h all animals were killed by i.p. sodium barbiturate and samples of the following tissues taken from each animal and weighed: eyelids (~20% of which is Meibomian gland), Zymbal's gland (ear canal: Pohl & Fouts, 1983), preputial gland, liver, oesophageal epithelium, footpads, KHT tumour, lung, muscle (quadriceps), kidney, brain (one

Correspondence: L.M. Cobb.
Received 10 April 1990; and in revised form 28 June 1990.

whole side), spleen, parotid salivary gland, blood, labial gland (lingual surface of cheek). The labial gland is an aggregation of sebaceous glands associated with the hairs in the labial vestibule (Quay, 1965).

Tritiated water was removed from the tissue samples by three cycles of dehydration with ethanol (overnight in the first instance and subsequently for a minimum of 4 h) and rehydration with distilled water. It was expected that this would remove tritiated water and some small water-soluble molecules (Franko et al., 1989). Blood was similarly dehydrated using three cycles of drying/rehydration *in vacuo* over $P_2O_5$. The samples were then prepared for liquid scintillation counting by dissolution in 1.0 ml of OptiSolve (LKB Scintillation Products (Pharmacia), Milton Keynes, UK) at 55°C for 16 h. Fifteen ml of OptiPhase (LKB Scintillation Products (Pharmacia), Milton Keynes, UK) was added and the samples counted on a Beckmann LS 5000 CE liquid scintillation counter (Beckman Instruments Inc., Fullerton, CA, USA) using the external standard generated H number to estimate the counting efficiency.

The following tissues were taken for ARG: eyelids, eyeball, labial gland, Zymbal's gland, preputial gland, KHT tumour, lung (to include bronchi), liver, oesophagus, muscle (quadriceps), kidney, stomach (squamous and glandular), brain (sagittal section), footpads, spleen, parotid gland and rostral part of the nasal passages. The tissues were processed to 3–5 μm paraffin sections and dipped in K2 emulsion (Ilford Emulsion, Knutsford, Cheshire, UK) for ARG. Exposure periods of from 1 to 14 weeks were used. The ARGs were developed, stained with haematoxylin and eosin, and examined microscopically to identify the distribution and density of grains in the emulsion overlying the tissue.

### Results

### *Tissue distribution studies*

The following results expressed as per cent injected tritium per g wet weight are multiplied by 100 for ease of presentation. For PIMO and RSU 1069 *respectively* the values (± s.d.) were: KHT tumour 7.8 (0.9), 33.0 (13.0); blood 1.2 (0.6), 33.6 (4.7); footpads 4.3 (1.1), 40.5 (4.1); parotid gland 3.2 (0.8), 21.6 (5.5); eyelid 20.5 (3.9), 171.9 (42.7); Zymbal's gland 3.6 (0.8), 22.8 (2.6); labial gland 5.5 (1.1), 62.8 (15.4); brain 1.3 (0.3), 18.5 (1.4); preputial gland 6.8 (2.9), 27.7 (11.8); liver 20.0 (5.4), 115.1 (25.1); spleen 3.5 (2.2), 43.3 (5.9); kidney 6.5 (1.4), 88.2 (9.9); oesophageal epithelium 34.2 (1.4), 99.4 (35.6); lung 3.1 (1.1), 32.2 (1.1); muscle 1.7 (0.3), 34.9 (3.5). The tissue measurements were then expressed as a ratio to the KHT tumour value and subjected to statistical analysis. All significant values together with a number of non-significant background tissue values are given in Table I.

**Table I** Activity in various tissues 24 h after injection, expressed as a ratio to the tumour value

| | *RSU 1069* | *PIMO* | *MISO*[a] |
|---|---|---|---|
| Liver | 3.5 (0.72) *** | 2.6 (0.34) *** | 3.5 (0.41) *** |
| Meibomian gland[b] | 5.2 (1.12) *** | 2.6 (0.26) *** | 1.3 (0.47) * |
| Oesophageal epithelium | 3.1 (0.73) *** | 4.37 (0.83) *** | 9.8 (0.80) *** |
| Kidney | 2.7 (0.51) ** | 0.83 (0.12) | 1.2 (0.35) |
| Labial gland | 1.9 (0.41) * | 0.71 (0.07) | n.a. |
| Blood | 1.0 (0.20) | 0.16 (0.03) | 0.49 (0.30) |
| Muscle | 1.1 (0.20) | 0.22 (0.02) | 0.38 (0.08) |
| Spleen | 1.3 (0.25) | 0.45 (0.13) | 0.40 (0.10) |

[a]Values from Cobb et al. (1990b). These values are for $^{14}C$-labelled MISO injected into mice bearing the A 110 mouse lung tumour. n.a., not available; [b]The eyelids, of which ~20% is Meibomian gland, were removed and the activity measured. ARGs showed that the Meibomian gland held much the greater part of the retained activity. The statistical significance of the tissue levels above tumour level was calculated using the Mann–Whitney test. *$P<0.05$; **$P<0.01$; ***$P<0.001$. Each compound had been injected into a group of five mice. s.d. in parentheses.

Also in this table are measurements taken from a previous publication on MISO (Cobb et al., 1990b).

### *Autoradiography*

With the exception of cells of macrophage lineage and melanin-containing cells (see below) the tissues at 24 h with noticeably high grain counts in PIMO- and RSU 1069-injected mice were the same as those in which high levels of MISO had previously been observed and measured (Cobb et al., 1989; Cobb & Nolan, 1989). They fell into five distinct tissues or groups of tissues.

1. Sebaceous gland group. This group comprised the sebaceous gland of the hair follicles, the Meibomian gland of the eyelids, Zymbal's gland of the ear canal, the labial gland on the lingual surface of the cheek and the preputial gland. These are histologically similar glands which all exhibit holocrine secretion; that is, the entire mature cell is secreted. Basal cells proliferate and a percentage mature to form the glandular secretion. The maturation process involves a gross enlargement of the cells by lipids, and nuclear degeneration. The radioactivity at 24 h was observed to some extent over the maturing cells and more so those in the process of secretion through the duct(s) to the exterior.

2. Keratinised stratified squamous epithelial cell group. This group comprised skin, oesophageal lining, the squamous area of the stomach lining and the footpads. The grains were concentrated over the stratum granulosum and the deeper parts of the keratinised layer (Figure 1). The highest grain density for both drugs (as for MISO) was in the oesophageal lining. The grain count in the skin of the eyelids, nares and footpads was patchy.

3. Lung. Activity was centred over the epithelium lining the airways. The grain density was clearly less in this tissue than in (1) and (2) above.

4. Liver. For both drugs, as for MISO, there was a high grain density in the cells surrounding the centrilobular veins (zone 3).

5. Parotid gland. As had previously been observed for MISO, PIMO and RSU 1069 showed a higher grain count over the epithelial cells lining the intralobular ducts than in the surrounding tissues.

**Figure 1** Junction of glandular with keratinised stratified squamous area of stomach (ARG). The PIMO metabolite 24 h post-injection is localised only in the squamous area, predominantly in the upper cells of the stratum pigmentosum and the adjacent lowest layers of the keratinised cells (arrow heads). One such area is shown inset enlarged × 3.5. At the bottom of the inset are the cells of the basal epithelium. They have a somewhat higher than background grain count; as do the cells of the next layer (stratum spinosum). The highest grain count is over the stratum granulosum and the first few layers of the keratinised cells. Thereafter the count falls away rapidly at the top of the inset. The bar represents 60 μm.

**Figure 2** Area of heavily pigmented skin to show binding of RSU 1069 to melanin (ARG). Twenty-four hours after the injection of $^3$H-RSU 1069 the ARG shows high activity over cells which are known to contain melanin (arrows). In this illustration only the grains in the ARG emulsion can be seen. The presence of melanin granules beneath required examination at high magnification when the black grains in the emulsion could be seen separately from the underlying pale golden brown melanin granules. The asterisk is in an area of heavy grain density over melanocytes and melanin-impregnated epidermal cells in the lower layers of the epidermis. The arrow head points to heavy grain density above a pigmented hair. The bar represents 60 $\mu$m.

In addition to the above tissues, PIMO- and RSU 1069-injected mice showed high grain density above melanin-containing cells (Figure 2). This was particularly clear in the skin of the eyelids and in the footpads. The melanin was in both melanocytes and adjacent melanin impregnated epithelial cells.

For RSU 1069 but not PIMO the macrophages in the liver (Kupffer cells), spleen and skin (tissue histiocytes) were associated with high grain density, indicative of the accumulation within these cells of a labelled metabolite and there was a high grain count over small cells in the brain which had a scattered distribution similar to that of microglia (macrophage lineage) (Merz *et al.*, 1987).

### Discussion

The main points arising from the results are that with the two MISO analogues PIMO and RSU 1069 activity was retained in much the same tissues as had previously been observed for MISO; and that there was activity in some normal tissues significantly in excess of the subcutaneous tumour.

The tissue samples were dehydrated because there is good evidence that the tritium associated with MISO tissue levels at 24 h is to a significant extent due to tritiated water (Franko *et al.*, 1989; Cobb *et al.*, 1990*b*). In the absence of any other evidence we have concluded that the same might apply to the two analogues of MISO and that with the tritiated water removed we believed that the activity was likely to be associated predominantly with bound metabolites of the two drugs. This concept is supported by the close correlation of high tritium retention with high grain count in the target tissues: sebaceous gland group, stratified squamous epithelium (oesophagus) and liver. In a recent publication on MISO we have pointed out that these tissues have in common high levels of reductase and we have suggested that, despite the likely presence of significant amounts of oxygen, nitroimidazoles are reduced to reactive, binding, metabolite(s) in these tissues (Cobb *et al.*, 1990*a*). The same may well apply to PIMO and RSU 1069.

The levels of radioactivity in the various tissues were measured at only one point in time (24 h) and it is possible that the ratio of one tissue level to another could change with time. However, the ARG grain density, which would reflect binding by reactive metabolites in the first few hours was high in tissues showing high tissue to tumour ratios at 24 h (Table I) thus indicating that the measurements of tissue activity at 24 h were probably not anomalous.

The high level of MISO retained by the liver has frequently been commented upon. Garrecht and Chapman (1983) injected $^{14}$C-MISO into mice bearing the EMT-6 tumour and reported high levels of adduct in liver and tumour as well as lower levels in all normal tissues sampled. The two drugs in the present report showed a similar high liver retention, with preferential localisation in the centrilobular zone (zone 3). Recently Maxwell *et al.* (1989*a*,*b*) and previously Van Os-Corby *et al.* (1987) have pointed to low oxygen tension being the likely cause of this retention for MISO. While this may be so there is also a higher reductase level in zone 3 which might contribute significantly to the localisation of all three drugs (Pette & Brandau, 1966; Cobb *et al.*, 1990*a*).

If it is true that high local reductase activity can outweigh the ability of oxygen to minimise the reduction of nitroimidazole by futile cycling there are clinical implications. For example, bioreductive cytotoxic drugs may be more effective in tumours which are not only hypoxic but have high reductase activity so that cytotoxic metabolites are produced in both hypoxic and normoxic regions. Also, where nitroimidazoles are used to identify hypoxic cells in pathological lesions by imaging techniques (e.g. NMR) they may also concentrate in normal tissues which have high levels of the appropriate reductase(s) and thus make interpretation more difficult.

The ARG grain localisation in part reflected the whole tissue measurements and supported the observation that some nitroimidazoles are able to form reactive, binding, metabolites in probably normoxic tissues with high reductase levels. It is by no means clear whether the high grain density can be regarded as a measure of cytotoxic potential in tissues. Cytotoxicity in the sebaceous gland group and the stratified squamous epithelial group would be difficult to recognise because the cells are normally discarded in a degenerate form from the skin. The position is different for the lung and liver where the cells observed to have a high grain count have a slow turn-over (replacement) rate. It is interesting to note that these same cells (airway epithelium and zone 3 of the liver) are damaged by CB 1954, a cytotoxin and radiosensitiser (Cobb, 1970) which has a similar electron affinity to MISO (Stratford *et al.*, 1981). It is thought that the reduction of CB 1954 to a cytotoxin is dependent on the 2 electron transfer reductase DT-diaphorase (Knox *et al.*, 1988).

In the present work it was noted that both PIMO and RSU 1069 were associated with melanin containing cells. This finding confirms previous observations that PIMO was associated with melanin in the eye in the mouse (Laurent *et al.*, 1989), with B 16 melanoma cells in the mouse (Walling *et al.*, 1989) and with xenografted human melanomas in nude mice (Lespinasse *et al.*, 1989). When Dische (1987) combined PIMO with radiation in patients with malignant melanoma he observed a favourable response compared with the expected response to radiation alone. The connection between the high grain density over melanin-containing cells in normal mouse tissue and the apparent improvement in response to radiation by patients with malignant melanoma is not clear to us. Many drugs adsorb to, or react with, melanin, and occasionally this leads to cytotoxic effects (Lindquist *et al.*, 1973). The presence of melanin in the uveal tract of the eye in pigmented mice and rats has not been observed to lead to toxic effect with PIMO or RSU 1069. In man the other critical tissue containing melanin is the brain (e.g. neuromelanin in the substantia nigra); however, melanin is not seen in the brain of mice or rats.

In conclusion, the accumulation of metabolites in normal tissues at levels greater than that for tumours is not restricted to MISO but occurs also in the analogues PIMO and RSU 1069.

We wish to thank Mr P. Webb and Dr M.D. Threadgill for the preparation of labelled PIMO and RSU 1069 (under NCI grant No. RO1-CA-44126), Dr I.J. Stratford for helpful discussions and Mr J. Bowler for implantation and husbandry of the tumour-bearing mice. The statistical analysis was carried out by Dr David Papworth.

### References

ADAMS, G.E., AHMED, I., SHELDON, P.W. & STRATFORD, I.J. (1984). Radiation sensitization and chemopotentiation: RSU 1069, a compound more efficient than misonidazole *in vitro* and *in vivo*. *Br. J. Cancer*, **49**, 571.

AHMED, I., JENKINS, T.C., WALLING, J.M. & 4 others (1986). Analogues of RSU-1069: radiosensitization and toxicity *in vitro* and *in vivo*. *Int. J. Radiat. Oncol. Biol. Phys.*, **12**, 1079.

ASQUITH, J.C., WATTS, M.E., SMITHEN, C.E. & ADAMS, G.E. (1974). Electron affinic sensitization. V. Radiosensitization of hypoxic bacteria and mammalian cells *in vitro* by some nitroimidazoles and nitropyrazoles. *Radiat. Res.*, **60**, 108.

CHAPMAN, J.D., FRANKO, A.J. & SHARPLIN, J. (1981). A marker for hypoxic cells in tumours with potential clinical applicability. *Br. J. Cancer*, **43**, 546.

COBB, L.M. (1970). Toxicity of the selective antitumor agent 5-aziridino-2,4 dinitrobenzamide in the rat. *Toxicol. Appl. Pharmacol.*, **7**, 231.

COBB, L.M. & NOLAN, J. (1989). Autoradiographic study of tritium labelled misonidazole in the mouse. *Int. J. Radiat. Oncol. Biol. Phys.*, **16**, 953.

COBB, L.M., NOLAN, J. & O'NEILL, P. (1989). Microscopic distribution of misonidazole in mouse tissues. *Br. J. Cancer*, **59**, 12.

COBB, L.M., HACKER, T. & NOLAN, J. (1990*a*). NAD(P)H nitroblue tetrazolium reductase levels in apparently normoxic tissues; a histochemical study correlating enzyme activity with binding of radiolabelled misonidazole. *Br. J. Cancer*, **61**, 524.

COBB, L.M., NOLAN, J. & BUTLER, S.A. (1990*b*). Tissue distribution of $^{14}C$- and $^{3}H$-labelled misonidazole in the tumour-bearing mouse. *Int. J. Radiat. Oncol. Biol. Phys.*, **18**, 347.

DISCHE, S. (1987). Radiotherapy using the hypoxic cell sensitizer Ro 03-8799 in malignant melanoma. *Radiother. Oncol.*, **10**, 111.

DISCHE, S., SAUNDERS, M.I., BENNETT, M.H. & 5 others (1986*a*). A comparison of the tumour concentrations obtainable with misonidazole and Ro 03-8799. *Br. J. Radiol.*, **59**, 911.

DISCHE, S., SAUNDERS, M.I., DUNPHY, E.P. & 5 others (1986*b*). Concentrations achieved in human tumours after administration of misonidazole, SR-2508 and Ro 03-8799. *Int. J. Radiat. Oncol. Biol. Phys.*, **12**, 1109.

FRANKO, A.J., RALEIGH, J.A., SUTHERLAND, R.G. & SODERLIND, K.J. (1989). Metabolic binding of misonidazole to mouse tissues. Comparison between labels on the ring and side-chain, and the production of tritiated water. *Biochem. Pharmacol.*, **38**, 665.

GARRECHT, B.M. & CHAPMAN, J.D. (1983). The labelling of EMT-6 tumours in BALB/C mice with $^{14}C$-misonidazole. *Br. J. Radiol.*, **56**, 745.

HORWICH, A., HOLLIDAY, S.B., DEACON, J.M. & PECKHAM, M.J. (1986). A toxicity and pharmacokinetic study in man of the hypoxic-cell radiosensitizer RSU 1069. *Br. J. Radiol.*, **59**, 1238.

KNOX, R.J., BOLAND, M.P., FRIEDLOS, F., COLES, B., SOUTHAN, C. & ROBERTS, J. (1988). The nitroreductase enzyme in Walker cells that activates 5-(aziridin-1-yl)-2,4-dinitrobenzamide (CB 1954) to 5-(aziridin-1-yl)-4-hydroxylamino-2-nitrobenzamide is a form of NAD(P)H dehyrogenase (quinone) (EC 1.6.99.2). *Biochem. Pharmacol.*, **37**, 467.

LAURENT, F., CANAL, P. & SOULA, G. (1989). Pharmacokinetics of Ro 03-8799 in mice bearing melanosarcoma: comparison with tumours without melanin. *Int. J. Radiat. Oncol. Biol. Phys.*, **16**, 1101.

LESPINASSE, F., THOMAS, C., BONNAY, M., MALAISE, E.P. & GUICHARD, M. (1989). Ro 03-8799: preferential relative uptake in human tumor xenografts compared to a murine tumor: comparison with SR-2508. *Int. J. Radiat. Oncol. Biol. Phys.*, **16**, 1105.

LINDQUIST, N.G. (1973). Accumulation of drugs in melanin. *Acta Radiol.*, suppl. 325.

MAXWELL, R.J., WORKMAN, P. & GRIFFITHS, J.R. (1989*a*). Demonstration of tumour-selective retention of fluorinated nitroimidazole probes by $^{19}F$ magnetic resonance spectroscopy *in vivo*. *Int. J. Radiat. Oncol. Biol. Phys.*, **16**, 925.

MAXWELL, A.P., MACMANUS, M.P. & GARDINER, T.A. (1989*b*). Misonidazole binding in murine liver tissue: a marker of cellular hypoxia *in vivo*. *Gastroenterology*, **97**, 1300.

MERZ, G.S., SCHWENK, V., SCHULLER-LEVIS, G., GRUCA, S. & WISNIEWSKI, H.M. (1987). Isolation and characterization of macrophages from scrapie-infected mouse brain. *Acta Neuropathol.*, **72**, 240.

PETTE, D. & BRANDAU, H. (1966). Enzym-Histogramme, und Enzymaktivitatsmuster der Ratternleber. *Enzymol. Biol. Clin.*, **6**, 79.

POHL, R.J. & FOUTS, J.R. (1983). Cytochrome P-450-dependent xenobiotic metabolizing activity in Zymbal's gland, a specialized sebaceous gland of rodents. *Cancer Res.*, **43**, 366.

QUAY, W. (1965). Comparative survey of the sebaceous and sudoriferous glands of the oral lips and angle in rodents. *J. Mammal.*, **46**, 23.

RALEIGH, J.A., FRANKO, A.J., TRIEBER, E.O., LUNT, J.A. & ALLEN, P.S. (1986). Covalent binding of a fluorinated 2-nitroimidazole to EMT-6 tumors in BALB/C mice: detection by F-19 nuclear magnetic resonance at 2.35 T. *Int. J. Radiat. Oncol. Biol. Phys.*, **12**, 1243.

RASEY, J.S., GRUNBAUM, Z., MAGEE, S. & 4 others (1987). Characterization of radiolabeled fluoromisonidazole as a probe for hypoxic cells. *Radiat. Res.*, **111**, 292.

RAUTH, A.M. (1984) Pharmacology and toxicology of sensitizers: mechanism studies. *Int. J. Radiat. Oncol. Biol. Phys.*, **10**, 1293.

ROBERTS, J.T., BLEEHEN, N.M., WALTON, M.I. & WORKMAN, P. (1986). A clinical phase I toxicity study of Ro 03-8799: plasma, urine, tumour and normal pharmacokinetics. *Br. J. Radiol.*, **59**, 107.

SAUNDERS, M.I., ANDERSON, P.J., BENNET, M.H. & 4 others (1984). The clinical testing of Ro 03-8799 pharmacokinetics, toxicology, tissue and tumor concentrations. *Int. J. Radiat. Oncol. Biol. Phys.*, **10**, 1759.

SMITHEN, C.E., CLARKE, E.D., DALE, J.A. & 4 others (1980). Novel (nitro-1-imidazolyl)-alkanolamines as potential radiosensitizers with improved therapeutic properties. In *Radiation Sensitizers*, Brady, L.W. (ed.) p. 22. Masson: New York.

STRATFORD, I.J., WILLIAMSON, C., HOE, S. & ADAMS, G.E. (1981). Radiosensitizing and cytotoxicity studies with CB 1954 (2,4-dinitro-5-azirinylbenzamide). *Radiat. Res.*, **88**, 502.

STRATFORD, I.J., WALLING, J.M. & SILVER, A.R.J. (1986). The differential cytoxocity of RSU 1069: cell survival studies indicating interaction with DNA as a possible mode of action. *Br. J. Cancer*, **53**, 339.

STRATFORD, I.J., ADAMS, G.E., GODDEN, J. & HOWELLS, N. (1989). Induction of tumour hypoxia post-irradiation: a method for increasing the sensitizing efficiency of misonidazole and RSU 1069 *in vivo*. *Int. J. Radiat. Biol.*, **55**, 411.

VAN OS-CORBY, D.J., KOCH, C.J. & CHAPMAN, J.D. (1987). Is misonidazole binding to mouse tissues a measure of cellular $pO_2$? *Biochem. Pharmacol.*, **36**, 3487.

VARGHESE, A.J., GULYAS, S. & MOHINDRA, J.K. (1976). Hypoxic-dependent reduction of 1-(2-nitro-1-imidazoyl)-3-methoxy-2-propanol by Chinese hamster ovary cells and KHT tumor cells *in vitro* and *in vivo*. *Cancer Res.*, **36**, 3761.

WALLING, J.M., DEACON, J., HOLLIDAY, S. & STRATFORD, I.J. (1989). High uptakes of RSU 1069 and its analogues into melanotic melanomas. *Cancer Chemother. Pharmacol.*, **24**, 28.

WEBB, P. & THREADGILL, M.D. (1990). Labelled compounds of interest as antitumour agents. Part II (i). Synthesis of [$^{2}H$] and [$^{3}H$] isotopomers of RSU 1069 and Ro-03-8799 (pimonidazole). *J. Labelled Compound Radiopharm.*, **28**, 257.